# Psychopathy by U.S. state: A translation of regional measures of the Big Five personality traits to regional measures of psychopathy

**DOI:** 10.1016/j.heliyon.2019.e01306

**Published:** 2019-03-18

**Authors:** Ryan H. Murphy

**Affiliations:** Southern Methodist University, The O'Neil Center for Global Markets and Freedom, SMU Cox School of Business, P.O. Box 750333, Dallas, TX, 75275, United States

**Keywords:** Psychology

## Abstract

Rentfrow et al. (2013) constructs a cross-section of the “Big Five” personality traits and demonstrates their relationship with outcomes variables for the continental United States and the District of Columbia. Hyatt et al. (Forthcoming) creates a means of describing psychopathy in terms of the Big Five personality traits. When these two findings are combined, a state-level estimate of psychopathy is produced. The estimate is conjectural, and if correct, it only describes the levels of psychopathy of states in relation of one state to one another, and is contingent on one particular conceptualization of psychopathy. Among the typical predictions made regarding psychopathy, the variable with the closest bivariate relationship with this new statistical aggregate is the percentage of the population in the state living in an urban area. There is no clear bivariate relationship of regional psychopathy with homicide, violent crime, or property crime rates.

## 1 Introduction

This paper makes a small contribution to geographical psychology by producing estimates of the level of psychopathy for each of the contiguous 48 U.S. states and the District of Columbia. Psychopathy, one of the “dark triad” of personality characteristics predicting antisocial behavior (Paulhus and Williams, 2002), is an important concept in psychology relevant for all social sciences. Combining recent research restating the triarchic model of psychopathy in terms of the Big Five personality characteristics with state-level data on personality, this paper provides a cross-section of 49 observations.

While a very small percentage of individuals in any given state may actually be true psychopaths, the level of psychopathy present, on average, within an aggregate population (i.e., not simply the low percentages of psychopaths) is a distinct research question. As in the case of geographical psychology more broadly, a measure of psychopathy by region facilitates testing a wide array of hypotheses, most notably with potential use in the interdisciplinary field of regional science. Although empirical operationalizations of psychopathy frequently treat it as a binary categorization, the Hare Psychopathy Checklist, Revised (PCL-R) (Hare, 1991) treats it as a spectrum. The operationalization of psychopathy found here is consistent with psychopathy as thought of as a spectrum.

Skeem et al. (2011) provide a complete overview of the psychopathic personality literature up to that point in time. Earlier, Hart and Hare (1994) and Lynam et al. (2005) investigated relationships between psychopathy and the Big Five personality test. More recently, Miller and Lynam (2012) perform a meta-analysis of findings regarding an alternative measure of psychopathy, the Psychopathic Personality Inventory (Lilienfeld and Widows, 2005), including its relationship with Big Five personality traits. Many have also investigated psychopathy and the Big Five in the context of the dark triad (Lee and Ashton 2005, 2014; Vernon et al., 2008; Hodson et al., 2009).

This paper builds on Rentfrow et al. (2013), who estimate the regional differences of the Big Five personality traits across the 48 contiguous states and Washington, D.C. The authors use five separate samples to develop a single estimate of each of the five traits for the regions, and examine the traits' relationship with various socioeconomic outcomes. They then use cluster analysis to identify three clusters of personalities – “Friendly and Conventional,” which roughly corresponds to the Midwest and the South, “Relaxed & Creative,” which is primarily found in the Southwest and Pacific Northwest, and “Temperamental & Uninhibited,” corresponding to the Northeast plus Texas.

Direct predecessors of Rentfrow et al. (2013) in constructing regional measures of personality include Plaut et al. (2002) and Krug and Kulhavy (1973). Later, notably, Elleman et al. (2018) has developed a longitudinal element to the literature, although the data that will remain the focus of this paper will be the cross-section developed by Rentfrow et al. (2013). We will return to this point later, following the initial exploration.

Other findings of geographical psychology are summarized in reviews by Rentfrow and Jokela (2016) and Rentfrow (2014). Variations in personality across the United States explain social capital, political orientation, and health (Rentfrow, 2010), as well as varied social measures like emotional health (McCann, 2011), suicide rates (Voracek, 2009), and entrepreneurship (Obschonka et al., 2015). Rentfrow et al. (2008) conceptualize how these interregional differences may arise theoretically. Elsewhere, Murray and Schaller (2014) explain how differentials in pathogen prevalence causes the variations to arise, and Jokela (2014) explains variations in terms of the willingness to migrate across different personalities.

This paper uses the Rentfrow et al. (2013) state-level data on personality in conjunction with Hyatt et al. (Forthcoming), who translate the Big Five personality traits into psychopathy (c.f. Widiger and Lynam, 1998; Miller et al., 2001). These latter authors argue counter to Patrick et al. (2009), who previously conceptualized psychopathy in a triarchic model - a constellation of disinhibition, boldness, and meanness. While Donnellan and Burt (2016) already explored the relationships between disinhibition, boldness and meanness and the Big Five personality traits, Hyatt et al. (Forthcoming) demonstrate that the triarchic model is actually nested within the Big Five. Boldness corresponds to low neuroticism and high extraversion, meanness corresponds to low agreeableness, and disinhibition corresponds to low conscientiousness. The findings of Rentfrow et al. (2013) and Hyatt et al. (Forthcoming) can thereby be combined into a method of estimating the level of psychopathy for each U.S. state.

While this may be an indirect methodology, and some amount of noise will inevitably be captured in the results, it is far less costly to re-estimate than the obvious alternative, i.e., attempting to implement the Hare Psychopathy Checklist (Hare, 1991) for each individual state. Previous estimates pertain primarily to prison populations (Hobson and Shine, 1998; Cooke, 1995; Rasmussen et al., 1999), or how psychopathy differs across cultures (e.g., Cooke, 1998). To our knowledge, there is no previous cross-sectional subnational data set on psychopathy.

The novelty of the findings is the greatest motivation for this exercise, and there is reason for considering the findings with caution. Hyatt et al. (Forthcoming) present evidence that the elements of the triarchic model can be reasonably well-explained by dimensions of Big Five personality traits, but less well-grounded is the subsequent connection between those dimensions and psychopathy itself. This is further complicated by the apparent lack of agreement between some measures of psychopathy (Sandick et al., 2012; Tew et al., 2015) and by the interpretation of multidimensional, continuous criteria of psychopathy, given the evidence of psychopaths as a discrete class (Harris et al., 1994). The meaningfulness of the results found here is contingent on both the translation of Big Five personality traits into psychopathy and that psychopathy is something that can be conceptualized as a statistical aggregate across people. And if the estimates are conceptually meaningful, the question remains of whether the size of the differences across regions is practically significant. The weak relationships found in the data can themselves be interpreted as support for skepticism, but whether that interpretation is correct requires further research beyond the scope of the presentation of this methodology and results.

To explore the data, this paper takes these estimates and compares them to four variables that relate to psychopathy in conventional, disaggregated data, with U.S. state as the unit of observation - homicide rate, violent crime rate, property crime rate, and the percentage of the state living in an urban area. It also uses information on the nine professions positively correlated with psychopathy and eight negatively correlated with psychopathy, according to Dutton (2012: 162). The bivariate relationships at the macro level are inconsistent and inconclusive as a whole, but certain bivariate relationships are statistically strong. Descriptively, for example, there is a strong correlation between psychopathy and the variable for percent urban.

## 2 Methods

Data for the Big Five personality traits for the 48 contiguous state plus the District of Columbia appear in the appendix of Rentfrow et al. (2013). Each of the Big Five receives a *T*-score centered on a mean of 50 and a standard deviation of ten. To apply Hyatt et al. (Forthcoming) and to create a raw score for psychopathy, a metric is created in which extraversion enters positively, while neuroticism, agreeableness, and conscientiousness enter negatively. Because extraversion and neuroticism are both intended to reflect “boldness,” they are each given a half weight.^1^ Mathematically, the metric is given as,

Psychopathy=(0.5)∗Extraversion−(0.5)∗Neuroticism−Agreeableness−Conscientiousness

This raw score appears in Table 1. The table then lists the standardized value of each region, followed by its rank among the 49. The top five observations in psychopathy are the District of Columbia, Maine, Connecticut, New York, and Maryland. The states that are least psychopathic are North Carolina, Tennessee, Mississippi, Nebraska, and South Carolina. Descriptive statistics for psychopathy can be found in Table 2.

**Table 1:** Psychopathy by state.

State	Raw Score	Z-Score	Rank
Alabama	-104.8	-0.31	33
Arizona	-98.75	0.08	19
Arkansas	-96.85	0.21	17
California	-86.05	0.91	10
Colorado	-100.8	-0.05	22
Connecticut	-70.70	1.92	3
Delaware	-83.00	1.11	7
District of Colombia	-53.9	3.02	1
Florida	-103.35	-0.22	29
Georgia	-116.2	-1.06	44
Idaho	-99.15	0.06	21
Illinois	-93.55	0.42	14
Indiana	-111.6	-0.76	37
Iowa	-101.95	-0.13	26
Kansas	-101.45	-0.09	25
Kentucky	-103.95	-0.26	31
Louisiana	-98.80	0.08	20
Maine	-70.20	1.95	2
Maryland	-81.90	1.19	5
Massachusetts	-82.60	1.14	6
Michigan	-104.40	-0.29	32
Minnesota	-109.35	-0.61	35
Mississippi	-120.60	-1.35	47
Missouri	-112.80	-0.84	38
Montana	-118.35	-0.87	40
Nebraska	-118.00	-1.18	46
Nevada	-86.40	0.89	11
New Hampshire	-102.30	-0.15	27
New Jersey	-86.35	1.07	9
New Mexico	113.50	-0.88	41
New York	-75.35	1.62	4
North Carolina	-128.90	-1.89	49
North Dakota	-102.40	-0.16	28
Ohio	-94.35	0.37	15
Oklahoma	-115.20	-1.00	43
Oregon	-109.20	-0.60	34
Pennsylvania	-98.60	-0.09	18
Rhode Island	-93.00	-0.46	13
South Carolina	-117.85	-1.17	45
South Dakota	-101.20	-0.08	23
Tennessee	-127.45	-1.80	48
Texas	-101.25	-0.08	24
Utah	-111.20	-0.73	36
Vermont	-112.85	-0.84	39
Virginia	-90.20	0.60	12
Washington	-103.95	-0.26	30
West Virginia	-114.75	-0.97	42
Wisconsin	-94.80	0.34	16
Wyoming	-83.15	1.10	8

**Table 2:** Descriptive statistics.

Variable	n	Mean	Std. dev.	Min	Max
Psychopathy	49	-100.003	15.26	-128.9	-53.9
Homicide Rate	49	5.188	3.050	1.3	18.5
Violent Crime Rate	49	387.637	178.716	123.8	1205.9
Property Crime Rate	49	2476.767	673.226	1512.9	4802.9
%Urban	49	73.912	14.929	38.7	100
Chief Executives	49	1.567	0.902	0.089	4.049
Lawyers	49	4.493	5.985	1.809	44.812
Media	48	0.273	0.136	0.122	0.934
Salespeople	49	60.996	8.528	21.258	81.577
Surgeons	44	0.344	0.182	0.076	0.94
Journalists	49	0.954	0.858	0.37	6.11
Police Officers	49	4.509	0.957	2.786	7.47
Clergy	47	0.347	0.401	0.105	2.6
Chefs	49	0.940	0.442	0.202	2.705
Care Aides	49	11.166	5.789	2.643	29.718
Nurses	49	22.563	3.566	15.663	32.126
Therapists	49	0.731	0.295	0.291	1.415
Beauticians	49	3.228	1.259	1.46	7.226
Teachers	49	36.993	5.331	19.371	49.336
Artists	49	3.691	1.051	2.068	7.054
Doctors	49	4.020	0.545	2.901	5.339
Accountants	49	8.457	2.231	4.903	15.769

NOTE: All occupations are stated in terms of per 1,000 jobs within the state.

Psychopathy is clustered in the Northeast and is loosely correlated with what Rentfrow et al. (2013) characterizes as the “Temperamental and Uninhibited Region,” which is defined in terms of “low extraversion, very low agreeableness and conscientiousness, very high neuroticism, and moderately high openness” (2013: 1008). This definition includes some positive relationships and some negative relationships with psychopathy, and unlike the “Temperamental and Uninhibited” cluster, Texas does not appear close to the top of the list for psychopathy.

The most extreme data point is the District of Columbia, which received a standardized score of 3.02. The next highest data point is Maine, which received a 1.95 standardized score. The presence of psychopaths in District of Columbia is consistent with the conjecture found in Murphy (2016) that psychopaths are likely to be drawn to and effective in the political sphere.^2^ Another point of interest is the odd placement of Wyoming (8^th^) relative to its geographic neighbors of Montana (40^th^), Idaho (21^st^), Colorado (22^nd^), Utah (36^th^), South Dakota (23^rd^), and Nebraska (46^th^). One possibility is that the sample size in Wyoming was the smallest of the 49 regions in Rentfrow et al. (2013) and this data point is simply incorrect, although Wyoming still had 3,166 observations.^3^

The clustering towards the top of the list is somewhat consistent with either a concentration of psychopaths in the Northeast and the Mid-Atlantic, or a concentration of psychopaths within urban areas. Maine, ranking the highest of any actual state (setting the District of Columbia aside) is a powerful data point in favor of the former hypothesis, but the presence of New Hampshire (27^th^) and Vermont (39^th^) far further down the list is evidence to the contrary.^4^

The four socioeconomic variables chosen through which to compare the psychopathy data are the homicide rate, the violent crime rate, the property crime rate in 2016 (see FBI, 2017), and the percentage of the state's population living in an urban area (U.S. Census Bureau, 2012). The link between psychopathy and criminal activity is a standard social scientific finding (see, e.g., Kiehl and Hoffman, 2011), whereas many bits of evidence suggest an allure of big cities for psychopaths (Geher, 2018). While population density is one operationalization of that latter hypothesis, the percentage living in an urban area is likely the better test.^5^

For additional candidates for correlates of psychopathy, we reference occupations that were found to be excessively likely or unlikely to be populated by psychopaths, as tabulated by Dutton (2012: 162). The occupations that were most disproportionately psychopathic were CEO, lawyer, media, salesperson, surgeon, journalist, police officer, clergyperson, chef, and civil servant. Those that were least psychopathic were care aide, nurse, therapist, craftsperson, beautician/stylist, charity worker, teacher, creative artist, doctor, and accountant. Although Dutton's list is what we make use of here, it should be noted that there is a broader academic literature on the industrial psychology of psychopaths, starting with Babiak (1995) and Babiak and Hare (2006) and continuing with a focus on corporate employees (Board and Fritzon, 2005; Babiak et al., 2010; Boddy, 2011). This literature has attempted to establish the preponderance of psychopaths in the corporate world and its implications, despite often facing severe data limitations.

Theoretically, an application of conventional economic treatments of labor markets is that, while an uncountable number of factors influence the geographic distribution of occupations, a population that is marginally more (less) psychopathic would express a greater (lesser) labor supply for these occupations than would otherwise occur, since they receive less (more) disutility from performing them relative to other occupations. This could operate through the initial occupational choices of those living in a given region, or via marginally more (less) psychopathic individuals moving to an area in response to greater (lesser) demand for the occupations located there.

Data by state for 2016 for occupations, expressed relative to a thousand of workers of a given state, can be found in the Occupational Employment Statistics (Bureau of Labor Statistics, 2018). Some of the occupations listed by Dutton correspond to a single occupation in the data set, while others correspond to different categories of occupations or sets of them. Three of these occupations, civil servant, charity worker, and craftsperson, do not have a clear correspondence to the data set and were dropped. Which of the data from Occupational Employment Statistics were used is provided in Table 3. Descriptive statistics for all variables are found in Table 2. We should note, finally, that for some states and occupations, no observations appear in the data. These states were dropped from the data, instead of appearing in the data as a zero.^6^

**Table 3:** Data definitions for employment categories listed by Dutton (2012), using occupational employment statistics from the Bureau of Labor Statistics.

Occupation	Occupation(s) or Occupational Category
Chief Executives	“Chief Executives”
Lawyers	“Lawyers”
Media	“Radio and Television Announcers” and “Broadcast New Analysts”
Salespeople	All occupations listed under 41-20, 41-30, and 41-40, minus “Counter and Rental Clerks,” plus “Demonstrators and Product Promoters,” “Real Estate Brokers,” “Real Estate Sales Agents,” and “Telemarketers”
Surgeons	“Surgeons”
Journalists	“Editors” and “Writers and Authors”
Police Officers	“Police and Sheriff's Patrol Officers”
Clergy	“Clergy”
Chefs	“Chefs and Head Cooks”
Care Aides	“Personal Care Aides”
Nurses	“Registered Nurses,” “Nurse Anesthetists,” and “Nurse Practitioners”
Therapists	“Psychiatrists” and “Clinical, Counseling, and School Psychologists”
Beauticians	All occupations listed under 39-50
Teachers	All occupations listed under 25-20 and 25-30
Artists	All occupations listed under 27-10
Doctors	“Pharmacists,” “Anesthesiologists,” “Family and General Practitioners,” “Internists, General,” “Obstetricians and Gynecologists,” and “Pediatricians, General”
Accountants	“Accountants and Auditors”

“Civil Servant,” “Charity Worker,” and “Craftsperson” were omitted due to lack of correspondence with any Occupational Employment Statistics occupational category.

Because of the constraints imposed by a limited sample size, the focus here will be to use the bivariate correlations to help in describing the nature of the data set, not to establish causality. In the section that will follow, the correlation coefficient *R* and the *t*-statistic from a simple regression using psychopathy as the sole explanatory variable will be given. This will be provided first with the full sample, and then with the District of Columbia omitted, given that for many of the variables, it drives much of the variation – almost to the exclusion of the other data points. This latter issue is a point of concern for the data set. Finally, following all this, an attempt at applying data from Elleman et al. (2018) to create longitudinal data for psychopathy is performed. But given the limits of this exploration, it is not warranted to think of it as the main result.

## 3 Results and discussion

Table 4 provides the simplified regression results of the four socioeconomic indicators and 17 occupations. The first column serves to remind the reader which direction the correlation is expected to run. These results are, at best, mixed. A few of the results move strongly and tightly in the expected direction, regardless of whether the District of Columbia is excluded, such as percent urban and the prevalence of lawyers. But this is not true of a majority of the relationships, including the relationship between psychopathy and the three classifications of crime rates. If the District of Columbia is excluded, property crime is actually strongly negatively correlated with psychopathy. The lack of a positive relationship between psychopathy and crime rates may be of broader interest for the social sciences in explaining trends in crime rates across the United States over time.

**Table 4:** Relationship of psychopath with variables of interest.

Variable	Expected Sign	COMPLETE SAMPLE		CENSOR D.C.	
		*R*	*t*	*R*	*t*
Homicide Rate	+	0.122	0.84	-0.229	-1.60
Violent Crime Rate	+	0.172	1.20	-0.183	-1.26
Property Crime Rate	+	-0.156	-1.08	-0.487	-3.78
%Urban	+	0.431	3.27	0.367	2.67
Chief Executives	+	0**.**094	0.65	-0.100	-0.68
Lawyers	+	0.509	4.05	0.451	3.43
Media	+	-0.153	-1.05	-0.244	-1.68
Salespeople	+	-0.283	-2.03	0.025	0.17
Surgeons	+	0.044	0.28	-0.027	-0.17
Journalists	+	0.546	4.46	0.369	2.69
Police Officers	+	0.135	0.93	-0.080	-0.55
Clergy	+	-0.116	-0.53	-0.092	-0.34
Chefs	+	0.439	3.35	0.383	2.81
Care Aides	-	0.046	0.32	-0.097	0.66
Nurses	-	-0.218	-1.53	-0.109	-0.74
Therapists	-	0.174	1.21	0.222	1.54
Beauticians	-	0.456	3.52	0.585	4.89
Teachers	-	-0.064	-0.44	0.189	1.31
Artists	-	0.424	3.21	0.275	1.94
Doctors	-	0.010	0.07	-0.115	-0.79
Accountants	-	0.488	3.83	0.352	2.55

More problematically, there are some pairs of regressions where the absolute values of the *t*-statistics are each greater than two while also achieving the non-hypothesized sign, these namely being the prevalence of accountants and beauticians (prevalence of artists just falls short of these cutoffs). One could readily formulate auxiliary explanations of why these relationships held in such a way, but that is not the point of this exercise. Rather, what seems to be the case is that the collective psychopathy of a region is a rather noisy indicator, at least bivariately. At the same time, given that for a few variables, the psychopathy measure achieves a *t*-statistic greater than four, there appears to be something underlying the correlation, regardless of whether the correlation is causal. As such, empirically, the measure of psychopathy by state is related to *something*, and is not simply a meaningless tangle of Big Five personality traits.

To summarize, there is a clear correspondence between psychopathy and percent urban, whether or not the District of Columbia is included. There is no correspondence between the prevalence of psychopaths and crime rates, although this question could be further addressed with a host of hypothetical control variables and denser longitudinal data than is presently available. A mixture of results is found using various professions, generally not supporting the hypothesis that psychopaths are prevalent in them, although this point is subject to quite a bit of interpretation. Results using limited longitudinal data, found in that which follow, are quite similar to what has been found initially.

Elleman et al. (2018) create the opportunity to apply somewhat more persuasive identification strategies by supplementing Rentfrow et al. (2013) with additional years of data. However, there are several points of caution to note before proceeding. First, while the title of Elleman et al. (2018) may suggest yearly data is readily available, the *new* data available used by Elleman et al. (2018) corresponds to two studies, one corresponding to the years 2006–2010 and the other to 2010–2015. Rentfrow et al. (2013) used five different overlapping samples (the years 1999–2005, 2005–2009, 2002–2009, 2008–2010, and 2007–2008) to construct their single cross-section. Furthermore, Elleman et al. (2018) report that they do not replicate the findings of Rentfrow et al. (2013) for both extroversion and agreeableness. On the other hand, one benefit of the Elleman et al. (2018) data is that it does include observations for Alaska and Hawaii, whereas Rentfrow et al. (2013) do not.

The two new cross sections from Elleman et al. (2018) are individually far smaller than the collective size of the samples merged by Rentfrow et al. (2013), so methodological problems concerning Rentfrow et al. (2013) would be more exaggerated in Elleman et al. (2018). Sample sizes by state in Elleman et al. (2018) range from 180 to 15,160, while sample sizes in Rentfrow et al. (2013) range from 3,166 to 177,085. To this point, Elleman et al. (2018) reports a significantly above average conscientiousness in North Dakota in the first sample and significantly below average conscientiousness in North Dakota in the second sample, a change over such a short period that it may be implausible.

Concerns with reliability of these sample sizes and the best strategy for operationalizing this data motivates not using it as a headline result. The sense from working with the data is that the approach of Rentfrow et al. (2013) of using truly large sample sizes to make geographic comparisons is appropriate, and this consideration should be a starting point for any extensions that would make even more granular comparisons or compare countries instead of U.S. states, whether that is concerning personality research of psychopathy specifically.

With all that said, Table 5 contains three sets of regressions which make use of the longitudinal element made possible using the extension by Elleman et al. (2018). Here, the imperfect solution settled on was to link the Rentfrow et al. (2013) data to the year 2005, the first Elleman et al. (2018) sample to 2010 and its second sample to 2015. Z-scores of psychopathy results using Elleman et al. (2018) and the Rentfrow et al. (2013) were used to place the measures on a common scale. For this analysis, we set aside Dutton's (2012) list of professions and focus on the hypotheses pertaining to crime (FBI, 2006; 2011, 2016) and percent urban. However, the Census Bureau does not yet have percent urban for years after 2010, so population density (Census Bureau, 2018), was used instead.

**Table 5:** Results of application of panel methods.

**LHS:**	**Homicide Rate**	**Violent Crime Rate**	**Property Crime Rate**	**Psychopathy**
*Pooled OLS*				
Psychopathy	0.719 (0.653)	38.615 (24.942)	-14.631 (65.653)	
LN Population Density				0.172* (0.091)
Constant	4.938*** (0.314)	394.937*** (16.268)	2911.632*** (64.540)	-0.789 (0.428)
$R^{2}$	0.047	0.050	0.001	0.047
n	151	151	151	151

*Panel with Year and State Fixed Effects*				
Psychopathy	0.361 (0.284)	4.436 (4.681)	-34.432 (31.383)	
LN Population Density				6.755 (5.560)
Constant	5.359*** (0.169)	424.045*** (7.275)	3358.332*** (37.630)	-30.611 (25.269)
$R^{2}$	0.052	0.021	0.183	0.047
n	151	151	151	151
*Dynamic Panel with Year and State Fixed Effects*				

Lagged Psychopathy	0.132* (0.075)	3.824 (3.396)	-33.443 (23.418)	
Lagged LN Population Density				3.841 (4.637)
Lagged Dependent Variable	-0.185*** (0.037)	0.357*** (0.105)	-0.219** (0.089)	-0.325*** (0.077)
Constant	5.522*** (0.215)	233.915*** (44.915)	3637.439*** (299.946)	-17.474 (21.126)
$R^{2}$	0.744	0.935	0.089	0.008
n	100	100	100	100

The three sets of regressions are four applications of pooled OLS, four applications of state and year fixed effects, and four dynamic panels with state and year fixed effects. The first three regressions of each group are concerned with crime, with psychopathy as the independent variable. The final regression in each group uses psychopathy as the dependent variable and logged population density as the independent variable. All regressions employ robust standard errors. Asterisks correspond to the convention of 10%, 5%, and 1% significance, and standard errors are reported parenthetically.

The results are modestly better for the hypothesis connecting crime to psychopathy, although the statistical relationships are still weak. Unsurprisingly, in the dynamic panel, which is only two periods because of the inclusion of a lag, no regression achieves any significance. The relationship between psychopathy and density remains (c.f. percent urban above) in terms of point estimates, as well as somewhat better *t*-statistics than in the crime regressions, but results are still weaker than what is found in the cross-sectional analysis prior.

The statistical results using longitudinal are qualitatively quite similar to what was found using the simple cross section. To some extent, this underlines either, that this research requires very large, expensive sample sizes to make workable longitudinal data very effective, or that the limits of the replication found in Elleman et al. (2018) deserve greater attention from the subfield of geographic psychology. This does not concern the question of the geographic distribution of psychopathy, however, except insofar as the analysis found here is predicated upon it.

## 4 Conclusion

Recent literature in psychology has studied the geographic distribution of various psychological characteristics. Using data from Rentfrow et al. (2013) and a methodology derived from Hyatt et al. (Forthcoming), we are able to derive state-level estimates for psychopathy. To our knowledge, these are the first subnational measures of psychopathy for the general population. It also differs from most empirical treatments of psychopathy; whereas most treatments view psychopathy as a binary question to be expressed as percentages of a population, the aggregate numbers created in this paper are closer to psychopathy as a spectrum, which is actually consistent with Hare (1991).

Areas of the United States that are measured to be most psychopathic are those in the Northeast and Mid-Atlantic. The least psychopathic are predominantly rural areas, both in the South and the West. The District of Columbia is measured to be far more psychopathic than any individual state in the country, a fact that can be readily explained either by its very high population density or by the type of person who may be drawn to a literal seat of power (as in Murphy, 2016). Additionally, Wyoming is an odd data point, ranking high in psychopathy given its place in the country and its lack of population. The inclusion of Maine along with the high population areas of the United State support the interpretation that psychopathy is clustered around the Northeast and not just population centers, although Vermont and New Hampshire contradict this interpretation. As a practical matter, it is recommended that empirical analysis making use of this data excludes the District of Columbia as a robustness check in some specifications.

Given that findings in this paper concern an indirect measure of psychopathy, the weight given to the empirical exploration as “tests” of the relationships between psychopathy and what it is thought to be associated with ought to be minimal. The purpose of the exploration was not to challenge previous findings, but to understand whether the conventional associations mapped to regional aggregates. Of the occupational and socioeconomic variables considered, psychopathy at the state level did not always correlate, or even relatively frequently correlate, with variables in the expected direction. The lack of correlation includes all three measures of crime, all of which actually enter negatively when the District of Columbia is excluded from the sample. Numerous explanations could be given for these results, but the fact will remain that it is too noisy of an indicator to reliably behave as expected in the bivariate context.

Still, that several regressions achieved such large *t*-statistics in small sample sizes suggests that this methodology is measuring an actual underlying signal. Additional waves of surveys of the Big Five personality traits by state (of sufficient sample size) would be what is needed to generate denser longitudinal data by state. Other extensions could develop similar subnational measures of psychopathy for already existing Big Five personality trait data in Britain (Rentfrow et al., 2015), Germany (Fritsch et al., 2018), and Switzerland (Götz et al., 2018). Ultimately, if longitudinal data were to be generated of a sufficient length of time, more credible empirical investigations of causality regarding the macro socioeconomic effects of psychopathy, conceived through the lens of geographic psychology, could be performed.

## Declarations

### Author contribution statement

Ryan H. Murphy: Conceived and designed the experiments; Performed the experiments; Analyzed and interpreted the data; Contributed reagents, materials, analysis tools or data; Wrote the paper.

### Funding statement

This research did not receive any specific grant from funding agencies in the public, commercial, or not-for-profit sectors.

### Competing interest statement

The authors declare no conflict of interest.

### Additional information

No additional information is available for this paper.

## Footnotes

1 A previous version of this paper gave extraversion and neuroticism a full weight, which causes modest differences in findings.

2 It also in some sense echoes the findings of Lilienfeld et al. (2012) on the relationship between psychopathic traits and presidential success. Joly et al. (Forthcoming) find that low levels of agreeableness correlate with political success.

3 More than one commenter has suggested that the high prevalence of psychopaths in Wyoming could be explained by Jackson Hole, due to the presence and nature of retreats taking place there.

4 Using a dummy variable set to 1 for the region of New England plus New York and New Jersey, both percent urban and this regional dummy are statistically significant in a multivariate regression explaining psychopathy. This exercise is not meant to suggest causality runs in the opposite direction as suggested elsewhere in this paper, but to note that each of these relationships can be statistically distinguished from one another.

5 Consider, for example, New York State. The urbanity of the environment for most living in New York is captured by the metric, whereas population density over-weights the small number of people living in the very rural areas of upstate New York. We will be forced to use population density later, however.

6 This affected three variables. For Media, Delaware is dropped. For Clergy, Utah and Wyoming and dropped. For Surgeons, Idaho, Maine, Mississippi, Utah, and West Virginia are dropped.
