# Corrigendum to “Psychopathy by U.S. state: A translation of regional measures of the Big Five personality traits to regional measures of psychopathy” [Heliyon 5 (3) (March 2019) e01306]

**DOI:** 10.1016/j.heliyon.2019.e01861

**Published:** 2019-06-11

**Authors:** Ryan H. Murphy

**Affiliations:** Southern Methodist University, The O'Neil Center for Global Markets and Freedom, SMU Cox School of Business, P.O. Box 750333, Dallas, TX, 75275, United States

Table 1 contains the following incorrect datapoints: In the "raw score" column for Montana, where it reads "-118.35," it should read "-113.35". In the "raw score" column for New Jersey, where it reads "-86.35," it should read "-83.65". In the "raw score" column for New Mexico, where it reads "113.50," it should read "-113.50". In the "z-score" column for Pennsylvania, where it reads "-0.09," it should read "0.09". In the "raw score" column for Virginia, where it reads "-90.20," it should read "-90.80". The author apologizes for the mistake. A corrected Table 1 is displayed below. This correction does not, in any way, compromise the findings of the study, either in terms of the methodology, results, or interpretations drawn from the data therein. Both the HTML and PDF versions of the article have been updated to correct the error.

**Table:** Table 1. Psychopathy by state.

State	Raw Score	Z-Score	Rank
Alabama	-104.8	-0.31	33
Arizona	-98.75	0.08	19
Arkansas	-96.85	0.21	17
California	-86.05	0.91	10
Colorado	-100.8	-0.05	22
Connecticut	-70.70	1.92	3
Delaware	-83.00	1.11	7
District of Colombia	-53.9	3.02	1
Florida	-103.35	-0.22	29
Georgia	-116.2	-1.06	44
Idaho	-99.15	0.06	21
Illinois	-93.55	0.42	14
Indiana	-111.6	-0.76	37
Iowa	-101.95	-0.13	26
Kansas	-101.45	-0.09	25
Kentucky	-103.95	-0.26	31
Louisiana	-98.80	0.08	20
Maine	-70.20	1.95	2
Maryland	-81.90	1.19	5
Massachusetts	-82.60	1.14	6
Michigan	-104.40	-0.29	32
Minnesota	-109.35	-0.61	35
Mississippi	-120.60	-1.35	47
Missouri	-112.80	-0.84	38
Montana	**-113.35**	-0.87	40
Nebraska	-118.00	-1.18	46
Nevada	-86.40	0.89	11
New Hampshire	-102.30	-0.15	27
New Jersey	**-83.65**	1.07	9
New Mexico	**-113.50**	-0.88	41
New York	-75.35	1.62	4
North Carolina	-128.90	-1.89	49
North Dakota	-102.40	-0.16	28
Ohio	-94.35	0.37	15
Oklahoma	-115.20	-1.00	43
Oregon	-109.20	-0.60	34
Pennsylvania	-98.60	**0.09**	18
Rhode Island	-93.00	-0.46	13
South Carolina	-117.85	-1.17	45
South Dakota	-101.20	-0.08	23
Tennessee	-127.45	-1.80	48
Texas	-101.25	-0.08	24
Utah	-111.20	-0.73	36
Vermont	-112.85	-0.84	39
Virginia	**-90.80**	0.60	12
Washington	-103.95	-0.26	30
West Virginia	-114.75	-0.97	42
Wisconsin	-94.80	0.34	16
Wyoming	-83.15	1.10	8

